# The Mental Hospital Service: Action by the Medical Officers

**Published:** 1914-02-28

**Authors:** 


### THE MENTAL HOSPITAL SERVICE.

### Action by the Medical Officers.

The following statement, which has been issued by the Association of Assistant Medical Officers of Mental Hospitals, England and Wales, has been sent this week to all members of visiting committees of public mental hospitals in England and Wales :—

Remuneration.

The remuneration of assistant medical officers has for many years been inadequate for professional men, and has only hitherto been accepted because of the possibilities of promotion to the position of medical superintendent. These possibilities have yearly been diminishing, and now the majority of medical men entering the asylum service must look solely to their remuneration as assistant medical officers. This is chiefly explained by the fact that the rapid growth in the size of new asylums, and the enlargement of older existing buildings, has led to a disproportionate increase in the number of assistant medical officers as compared with that of medical superintendents. At the same time the work of assistant medical officers has become more responsible, requiring mature experience.

At the present time the remuneration of medical superintendents averages over £1,000 per annum, whilst that of assistant medical officers of five to ten years' lunacy experience averages about £360 per annum, and those over ten years' experience about £425 per annum (in each case value of emoluments is included). The Association of Assistant Medical Officers, after a mature consideration of the comparative advantages and remuneration of medical men of similar standing both in private practice as well as in other public services, holds it not unreasonable that senior assistant medical officers should receive a maximum of two-thirds that of the medical superintendent's maximum (the ratio of five-sevenths obtains in some foreign asylums). The Association is also of opinion that other assistant medical officers should similarly receive pay and emoluments increasing in a definite ratio according to their length of service as well as seniority, it being a well recognised fact that experience and efficiency do not necessarily ensure promotion. It is therefore felt that the time has come when an effort should be made to induce medical men to enter the asylum service by offering them adequate prospects which they can reasonably expect to realise.

Marriage and Accommodation.

It has been in most cases the practice of asylum committees to look upon their assistant medical officers as celibates. Such a condition was accepted so long as promotion to the post of medical superintendent, with consequent facilities for marriage, was not a remote contingency. The position no longer holds. Many assistant medical officers cannot have any reasonable assurance that they will rise above the level of senior or second assistant medical officer. They ought therefore to have the freedom of a normal life, a privilege which is granted in some instances to assistant medical officers, as well as to the other officers and members of the staff of asylums.

Apart from the question of marriage, it is not unreasonable that a medical man, after some years spent in the asylum service, should expect to be able to live in a separate house.

Medical Work in Asylums.

It is desired to draw your attention to the fact that, although the science of medicine has advanced with enormous strides of late years, there has been little corresponding advance in this country in psychiatry. Instruments and appliances for modern medical treatment, and the accommodation for such clinical laboratory work as is carried on in every general hospital, are too seldom provided; and the number of medical officers carrying on special work compares very unfavourably with the standard regarded as essential by modern medical opinion.

It is feared that the power given to committees of visitors to call upon members of their medical staff to retire after reaching a pensionable age has in too many cases fallen into desuetude.

Diploma in Psychiatry.

The necessity of keeping abreast of modern discoveries in psychiatry has now become so important that it will certainly soon be indispensable that assistant medical officers entering and rising in the service should take out special courses in the scientific branches of their work at one or other university, and it is suggested that study leave should be granted to assistant medical officers to enable them to do this, and to obtain one of the diplomas in psychiatry now granted by several universities.

The Association of Assistant Medical Officers has endeavoured to exclude from this statement any recommendations which would involve fresh legislation.

